# Compensatory Responses During Slip-Induced Perturbation in Patients With Knee Osteoarthritis Compared With Healthy Older Adults: An Increased Risk of Falls?

**DOI:** 10.3389/fbioe.2022.893840

**Published:** 2022-06-15

**Authors:** Xiping Ren, Christoph Lutter, Maeruan Kebbach, Sven Bruhn, Qining Yang, Rainer Bader, Thomas Tischer

**Affiliations:** ^1^ College of Physical Education and Health Sciences, Zhejiang Normal University, Jinhua, China; ^2^ Biomechanics and Implant Technology Research Laboratory, Department of Orthopedics, Rostock University Medical Center, Rostock, Germany; ^3^ Institute of Sport Science, Faculty of Philosophy, University of Rostock, Rostock, Germany; ^4^ Department of Joint Surgery, The affiliated Jinhua Hospital, Zhejiang University School of Medicine, Jinhua, China

**Keywords:** musculoskeletal disorders, older adults, treadmill-induced perturbation, slips and falls, compensatory step

## Abstract

**Background:** Functional impairment of the knee joint affected by osteoarthritis and loss of muscle strength leads to a significant increase in the number of falls. Nevertheless, little is known about strategies for coping with gait perturbations in patients with knee osteoarthritis (KOA). Thus, this study aimed to examine the compensatory strategies of patients with KOA in response to a backward slip perturbation compared with healthy older adults.

**Methods:** An automated perturbation program was developed by using D-Flow software based on the Gait Real-time Analysis Interactive Lab, and an induced backward slip perturbation was implemented on nine patients with severe KOA (68.89 ± 3.59 years) and 15 age-matched healthy older adults (68.33 ± 3.29 years). Step length, gait speed, range of motion, vertical ground reaction forces, lower extremity joint angles, and joint moments were computed and analyzed.

**Results:** Compared with older adults, patients with KOA had significantly lower step length, gait speed, and vertical ground reaction forces in both normal walking and the first recovery step following backward slip perturbations. Inadequate flexion and extension of joint angles and insufficient generation of joint moments predispose patients with KOA to fall. Hip extension angle and flexion moment, knee range of motion, and vertical ground reaction forces are key monitoring variables.

**Conclusion:** The risk of falls for patients with KOA in response to backward slip perturbations is higher. Patients with KOA should focus not only on quadriceps muscle strength related to knee range of motion but also on improving hip extensor strength and activation through specific exercises. Targeted resistance training and perturbation-based gait training could be better options.

## Introduction

Knee osteoarthritis (KOA) is a highly prevalent degenerative joint disease in older adults (Sharma, 2021), with an incidence that has doubled since the mid-20th century (Wallace et al., 2017). It poses major socioeconomic challenges throughout the world (Cross et al., 2014; Cui et al., 2020).

KOA commonly causes considerable pain (Neogi, 2013) and dysfunction (Alencar et al., 2007). In addition to the direct impairment, these deficiencies are also likely to cause falls during normal walking (Manlapaz et al., 2019; Deng et al., 2021). Falls by themselves can lead to a high burden of musculoskeletal diseases like fractures of the hip, distal radius, and proximal humerus, among others (Lamb et al., 2020). Considering patients with KOA, women with OA have a 25% higher risk of falls and a 20% higher risk of fracture than their peers without OA (Prieto-Alhambra et al., 2013). Moreover, an increase in the number of joints with symptomatic KOA of the lower extremities increases the risk of falling (Doré et al., 2015). Therefore, preventing falls among patients with KOA is a critical but often-overlooked topic. An in-depth understanding of the compensatory response to a slip is fundamental to help fall-prone populations reduce the incidence of falls (Marigold et al., 2003; Gholizadeh et al., 2019). To the best of our knowledge, there is only a limited understanding of compensatory strategies after gait perturbations in patients with KOA, especially for a slip-induced perturbation.

“Slip” is a type of external dynamic perturbation of walking, which can induce a sudden displacement of the base of support (BoS) into the anterior or posterior direction relative to the center of mass (CoM) (Debelle et al., 2020; Lee et al., 2020). Both a single session of slip perturbation training and single-repeated slip training have positive effects on improving dynamic stability (Pai and Bhatt, 2007; Lee et al., 2020). Humans can quickly regain stability and maintain balance from the same type of perturbation (Debelle et al., 2020). The “first-trial effect” has been used to describe the training effects of older adults experiencing the first slip perturbation, suggesting that first-exposure trials generate rapid adaptive effects, and such effects could be maintained for up to a full year (Pai and Bhatt, 2007; Liu et al., 2017). Subsequently, Inkol et al. (2019) found that the first response of a slip perturbation has the largest effect on the gait variables compared with the subsequent perturbations of the same type (Inkol et al., 2019), and the first step following the perturbation is the most important protective postural strategy (Maki and McIlroy, 1997; Melzer et al., 2008). Further, the step length of the first recovery step after a slip perturbation determines the stability of the subsequent steps and is corrected with balance recovery mechanics (Debelle et al., 2021). It has been found that the frequency of backward slip on the oiled surfaces is twice as high as that of the forward slip (Nagano et al., 2013), implying a high risk of falls, yet this perturbation is also most prevalent in daily life (Debelle et al., 2020). However, there is still a lack of research on the effects of backward slip perturbations in people at high risk of falls, such as patients with KOA, and it is unclear how the first step after the perturbation plays a protective role. Therefore, effective monitoring of the first step after a slip perturbation in patients with KOA is crucial.

Moreover, lower extremity joint kinematics and kinetics partially influence the outcome of perturbations and hence play a critical role in understanding the complicated link between gait and slip-induced falls (Karamanidis et al., 2020). The application of joint moments to clinical-pathological gait analysis, such as knee and hip OA, is of increasing interest (Lathrop-Lambach et al., 2014). For example, changes in the magnitude of knee joint moments indicate the progression and severity of OA development (Asay et al., 2018). The quadriceps strength is directly related to knee flexion moment (Lisee et al., 2019), and the medial-lateral load distribution is usually expressed in terms of knee adduction moments (Zeighami et al., 2021). However, few studies have addressed the effect of joint moment application in response to gait perturbations in patients with KOA.

Given the above-mentioned research gap, this study aimed to examine the compensatory strategies of patients with KOA and healthy older adults in response to backward slip perturbations, mainly focusing on comparing the differences and similarities between the first recovery step (Rec1) after the perturbation and the normal gait (Normal), and thus to determine whether patients with KOA are more prone to falls. We developed and implemented a procedure to automatically trigger a slip perturbation and then measure the effects of gait spatiotemporal parameters and the lower extremity kinematics and kinetics on the recovery of dynamic stability. We hypothesized patients with KOA are at higher risk of falls relative to healthy older adults and the protective strategy in the first step following the backward slip perturbation focuses on the effective compensatory effect of the lower extremity.

## Materials and Methods

### Subjects

Nine patients (age: 68.89 ± 3.59 years; body height: 1.69 ± 0.11 m; body mass: 97.53 ± 19.16 kg) with advanced left KOA with persistent pain [Kellgren and Lawrence (K-L) grade 4] and 15 age-matched healthy older adults (age: 68.33 ± 3.29 years; body height: 1.76 ± 0.10 m; body mass: 81.13 ± 13.99 kg) participated in this investigation. The dominant leg was right in all participants. The inclusion criteria for patients with KOA were those who were about to undergo total knee arthroplasty on the left knee within 3 months, and the inclusion criterion for older adults was no lower extremity history of disorders and injuries. Patients with KOA were recruited through the Rostock Orthopedic Clinic, and older adults were recruited from different communities. Written consent was obtained from all participants prior to the measurement. Ethical approval was granted by the committee of the Rostock University Medical Center, Germany (A2019-0231). All measurements were carried out in compliance with the Declaration of Helsinki.

### Experimental Protocol

**FIGURE 1:**
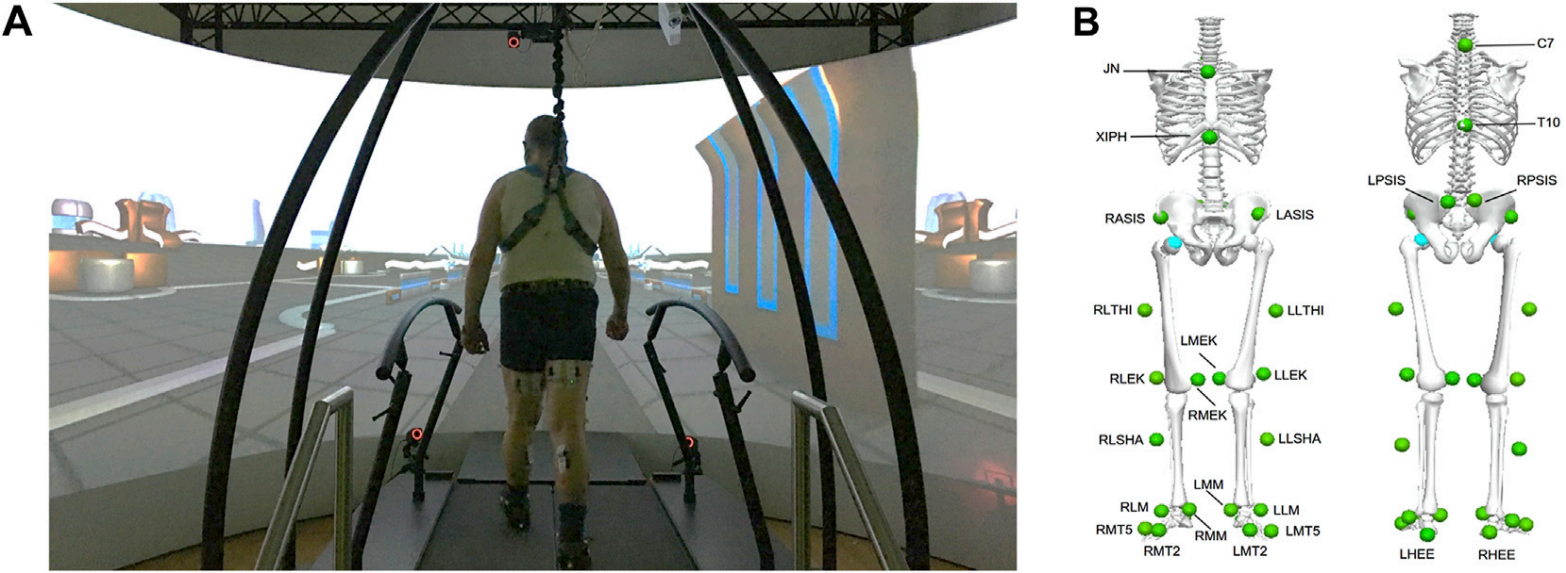
The Gait Real-Time Analysis Interactive Lab (GRAIL) and the industrial-style virtual reality scenario involved in this study **(A)**; front and rear view of marker set used in Human Body Model (v2) with its specific 26 markers (green), which involves the anatomical positions of the lower extremities and trunk **(B)**.

The Gait Real-time Analysis Interactive Lab (GRAIL) (Motek Medical B.V., Houten, Netherlands), which integrates multiple devices, was utilized for the investigation. A 10 infrared cameras motion capture system (Vicon Bonita B10, Vicon Metrics Ltd., Oxford, United Kingdom) was utilized to track the marker trajectories at 100 Hz, and an embedded treadmill force plate (ForceLink B.V., Culemborg, Netherlands) was used to record the ground reaction forces (GRFs) at 1,000 Hz. The D-flow software (v3.34, Motek Medical B.V.) synchronized all hardware and triggered signals for data collection. Participants felt like they were in an industrial-style virtual reality scenario during the entire measurement (Figure 1A).

Prior to the investigation, the subjects’ demographic parameters and leg length were recorded and the Timed Up and Go test (TUG) was performed. Subsequently, 26 reflective markers with a diameter of 1.4 cm (Figure 1B) were attached to the anatomical landmarks according to the Human Body Model (HBM, v2, Motek Medical B.V.). Self-selected speeds were obtained during the participants’ 6-min treadmill familiarization (Meyer et al., 2019; Oudenhoven et al., 2019). In the first trial, normal walking was collected for 2 min. In the second trial, right-side treadmill belt posterior acceleration (to induce a “slip-like” effect) and deceleration (to induce a “trip-like” effect) perturbations were performed (van den Bogaart et al., 2020). Two different types of perturbations were set up in each trial, which were randomly and automatically triggered by a custom program. The acceleration and deceleration intensities were set at 3 m/s^2^. The perturbation occurred and lasted for 300 ms after the speed reached a specific value, then returned to normal speed. All perturbations happened at the moment of the right heel strike. Each perturbation occurred at a pseudo-random interval of 15–20 s for a total of six times. There were sufficient breaks to prevent fatigue and knee pain. The whole session took around 15 min for each participant, and each participant successfully completed the measurements. During the entire session, each participant was protected from falling by a harness and was able to cope with the perturbation task and did not grasp the handrails of the treadmill.

### Data Processing

The Gait Offline Analysis Tool (GOAT, version 4.1, Motek Medical B.V.) (Oudenhoven et al., 2019; Bahadori et al., 2021) was used for data processing with a built-in HBM computational model, which had been verified previously (Van Den Bogert et al., 2013; Falisse et al., 2018; Flux et al., 2020). The local maximum of the anterior-posterior position of the heel marker relative to the pelvis was used to determine the heel-strike event (Zeni et al., 2008). A second-order low-pass Butterworth filter at 6 Hz was set to filter kinematic data, which were found to be the highest frequency in kinematics related to gait (Winter et al., 1974). To prevent artifacts, GRFs were processed with the same filter cutoff as for kinematics (Bisseling and Hof, 2006; Van Den Bogert et al., 2013). Inverse kinematics and inverse dynamics algorithms were conducted to calculate the gait spatiotemporal parameters, joint angles, and joint moments of the lower extremity by deploying HBM (Van Den Bogert et al., 2013). The joint moments were normalized to the participant’s body mass to reduce the confounding effect (Moisio et al., 2003). To compare continuous time series variables, each joint angle and moment was normalized to the percentage of the gait cycle (101 data points, 0%–100%).

For normal walking, 20–25 consecutive strides were averaged for each participant, an approach consistent with previous studies (Kribus-Shmiel et al., 2018; Kroneberg et al., 2019; Riazati et al., 2019). For slip-perturbed walking, only the slip_Rec1 of the first trial was used for analysis (Debelle et al., 2020). In the current study, the focus was on treadmill belt acceleration perturbations in the posterior direction, and only the participants’ recovery compensatory strategies in response to slip perturbations were analyzed. The following parameters were processed: step length, gait speed, vertical GRFs (vGRFs), and lower extremity joint angles, joint moments in the sagittal plane. It has been reported that anteroposterior gait perturbations mainly affect parameters in the sagittal plane (Yoo et al., 2019).

### Statistical Analyses

Before determining the type of statistical analysis, the normality of the data was assessed by using the Shapiro–Wilk test. Independent and paired sample *t*-tests were utilized for zero-dimensional data analysis of demographic characteristics, leg lengths, TUG scores, step length, gait speed, and peak vGRFs. One-dimensional time-series statistical analysis of joint angles and joint moments was conducted by using open-source statistical parametric mapping (SPM), which is based on random field theory (Pataky et al., 2015). All statistical analyses were performed by using GraphPad Prism (v8.0.2, GraphPad Software Inc., La Jolla, CA, United States) and Matlab 2018b (The MathWorks Inc., Natick, MA, United States). The significance level was set at *p* < 0.05.

## Results

### Basic Information of Participants

Demographic characteristics, leg length, and TUG score from the participants are presented in Table 1. Body mass (*p* = 0.0242), body mass index (BMI) (*p* = 0.0010), leg length (*p* = 0.0423), and TUG scores (*p* = 0.0053) were significantly different between the patients with KOA and older adults.

**TABLE 1:** Basic information of patients with knee osteoarthritis (KOA) and older adults.

Variables	Patients with KOA (*n* = 9; 4 females)	Older adults (*n* = 15; 4 females)	*p-*value
Age (years)	68.89 ± 3.59 [66.13, 71.65]	68.33 ± 3.29 [66.51, 70.15]	0.7020
Body height (m)	1.69 ± 0.11 [1.60, 1.77]	1.76 ± 0.10 [1.71, 1.81]	0.0989
Body mass (kg)	97.53 ± 19.16 [82.81, 112.30]	81.13 ± 13.99 [73.38, 88.88]	0.0242*
BMI (kg/m2)	34.03 ± 3.43 [31.39, 36.67]	26.24 ± 4.83 [23.56, 28.91]	0.0010**
Leg length (m)	0.86 ± 0.09 [0.79, 0.93]	0.93 ± 0.07 [0.89, 0.97]	0.0423*
TUG (s)	15.28 ± 3.96 [12.24, 18.32]	10.32 ± 1.23 [9.64, 11.00]	0.0053**

The data are presented as the mean ± standard deviation [95% Confidence Interval]. *p < 0.05, **p < 0.01. Abbreviations: BMI, body mass index; TUG, timed up and go test.

### Spatiotemporal Parameters and vGRFs

**FIGURE 2:**
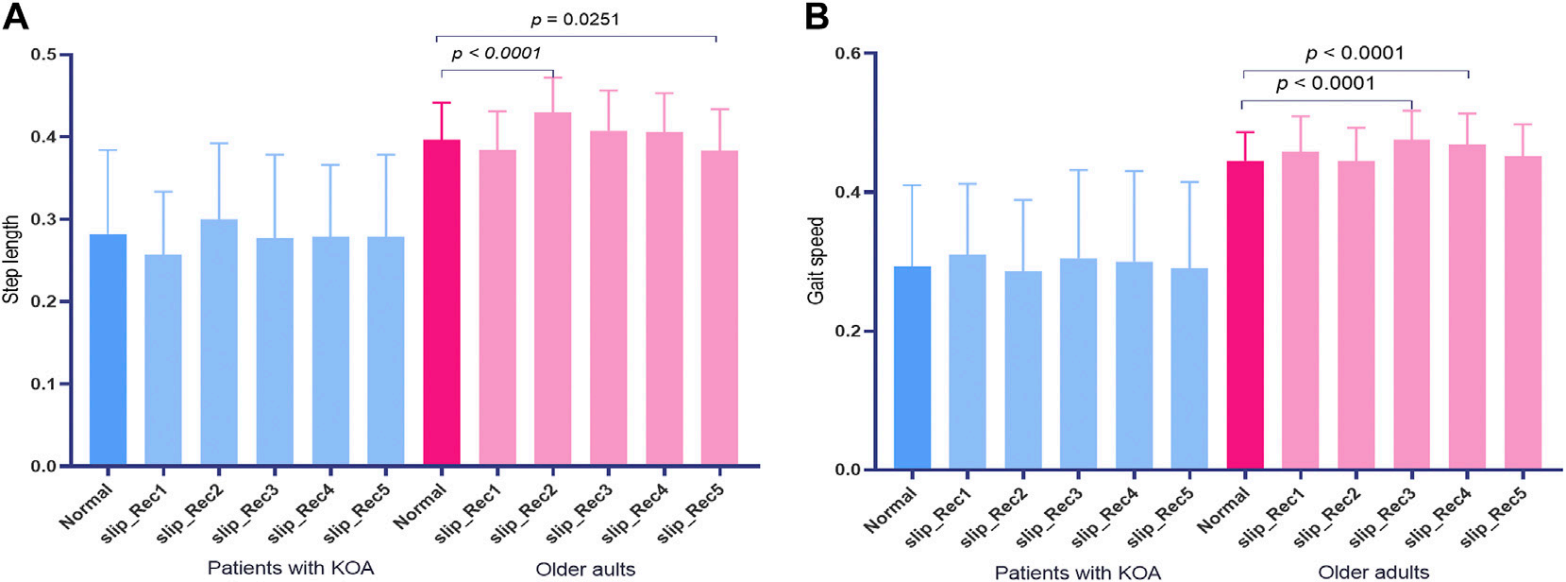
The step length **(A)** and gait speed **(B)** in normal walking (Normal) and the five steps following the perturbation. Blue bars indicate patients with knee osteoarthritis (KOA) and red bars indicate older adults.

The step length normalized to body height (BH) and gait speed normalized to leg length ( $\sqrt{gl_{0}}$ , with *g* = 9.81 m/s^2^, *l*_*0*,_ leg length) of Normal and the five consecutive steps following the perturbation are presented in Figure 2 **.** There were no changes in step length and gait speed in patients with KOA, while there were significant differences in step length between Normal and slip_Rec2 and slip_Rec5 (*p* < 0.0001 and *p* = 0.0251, respectively) (Figure 2A), and significant differences in gait speed between Normal and slip_Rec3 and slip_Rec4 (*p* < 0.0001 and *p* < 0.0001, respectively) (Figure 2B).

**FIGURE 3:**
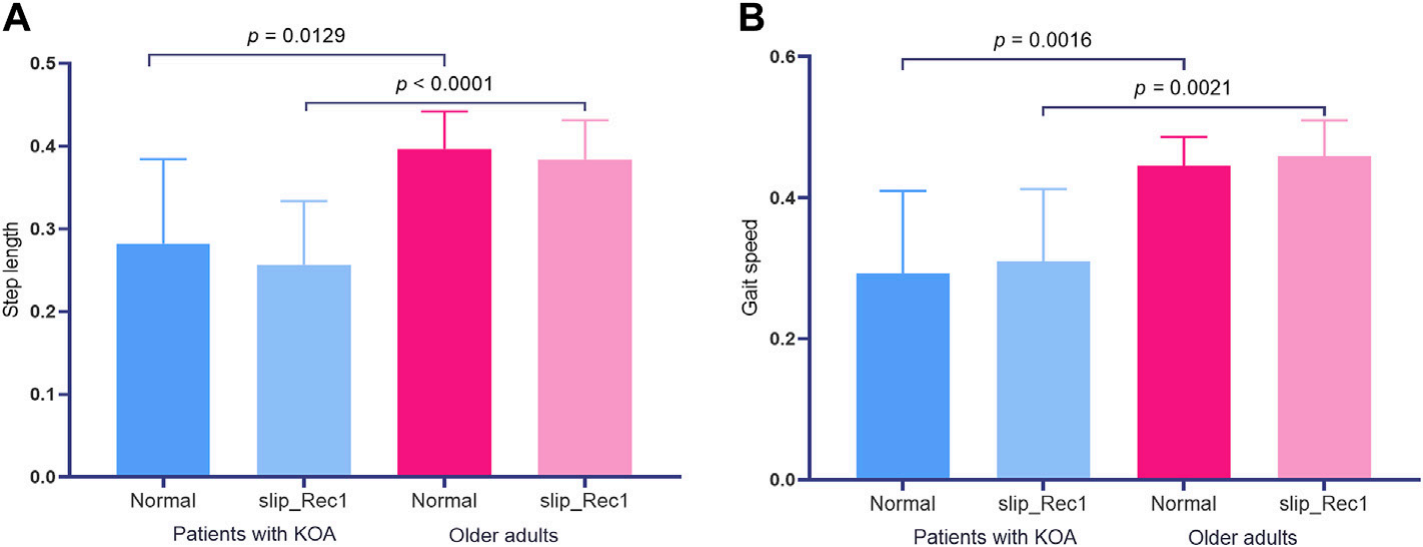
Zero-dimensional statistical analysis of step length **(A)** and gait speed **(B)** between Normal and slip_Rec1 among patients with knee osteoarthritis (KOA) and older adults.

The step length for Normal was 0.28 ± 0.10 [95% confidence interval (CI) 0.20, 0.36] and 0.40 ± 0.05 [95% CI 0.37, 0.42] for patients with KOA and older adults, while in the slip_Rec1 gait, the values were 0.26 ± 0.08 [95% CI 0.20, 0.32] and 0.38 ± 0.05 [95% CI 0.36, 0.41], respectively. There were no changes in the step length of Normal and slip_Rec1 in patients with KOA and older adults. There were significant differences in the step lengths of Normal (*p* = 0.0129) and slip_Rec1 (*p* < 0.0001) between patients with KOA and older adults (Figure 3A). The step length of patients with KOA was significantly smaller than that of older adults.

The gait speeds for Normal and slip_Rec1 between patients with KOA and older adults were 0.29 ± 0.12 [95% CI 0.20, 0.38] and 0.45 ± 0.05 [95% CI 0.42, 047] versus 0.31 ± 0.10 [95% CI 0.23, 0.39] and 0.46 ± 0.05 [95% CI 0.43, 0.49], respectively. There was no significant difference in the same group, whereas there were differences between the groups under the same condition (*p* = 0.0016 for Normal and *p* = 0.0021 for slip_Rec1) (Figure 3B). Patients with KOA had a significantly lower gait speed than older adults.

**FIGURE 4:**
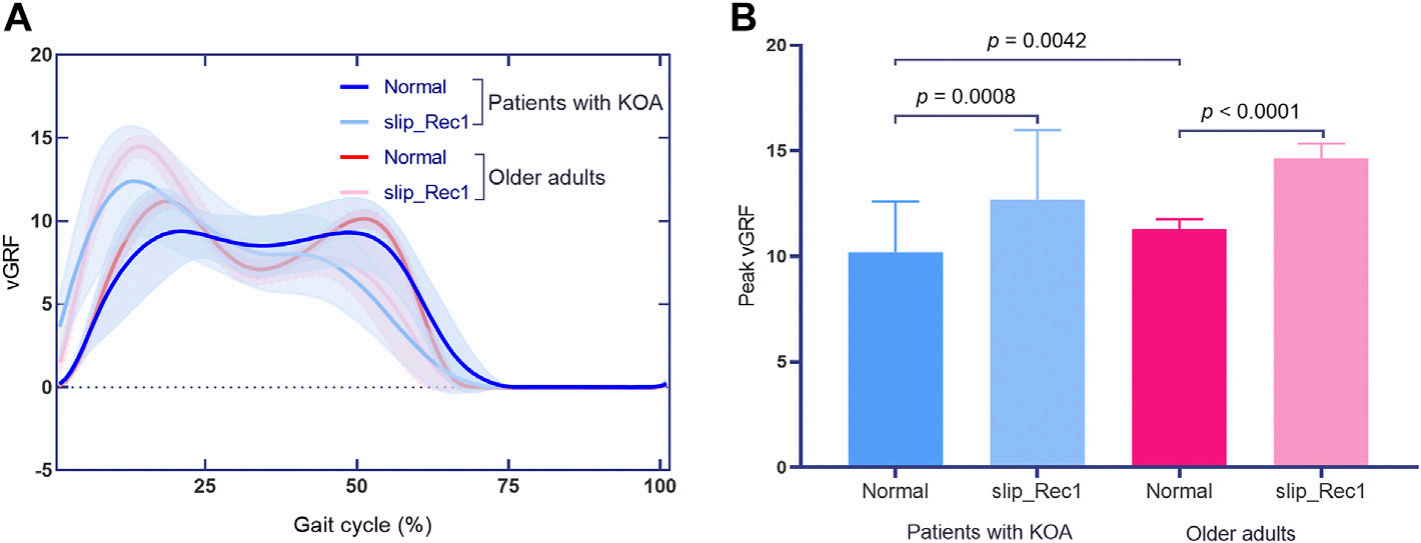
The vertical ground reaction force (vGRF) curves in Normal and slip_Rec1 **(A)**; zero-dimensional statistical analysis of peak vGRFs among patients with knee osteoarthritis (KOA) and older adults **(B)**.

The vGRF normalized to body mass (BM) for Normal and slip_Rec1 in patients with KOA and older adults during the gait cycle are presented in Figure 4A. The Normal and slip_Rec1 phases of vGRFs reaching zero in patients with KOA were 71% and 67%, respectively, while these values were 66% and 65% in older adults. Peak vGRFs for Normal and slip_Rec1 in the vertical direction for patients with KOA and older adults were 10.21 ± 2.38 [95% CI 8.38, 12.04] and 12.70 ± 3.29 [95% CI 10.17, 15.22] versus 11.30 ± 0.46 [95% CI 11.05, 11.55] and 14.64 ± 0.70 [95% CI 14.25, 15.02], respectively. There were significant differences between the two conditions in the same group (*p* = 0.0008 for patients with KOA and *p* < 0.0001 for older adults). The vGRFs significantly increased during slip_Rec1. The increases were 24.39% for patients with KOA and 29.56% for older adults. Patients with KOA showed a significantly smaller value than older adults in Normal (*p* = 0.0042) (Figure 4B). However, such a difference was not observed in slip_Rec1.

### Ankle Joint

**FIGURE 5:**
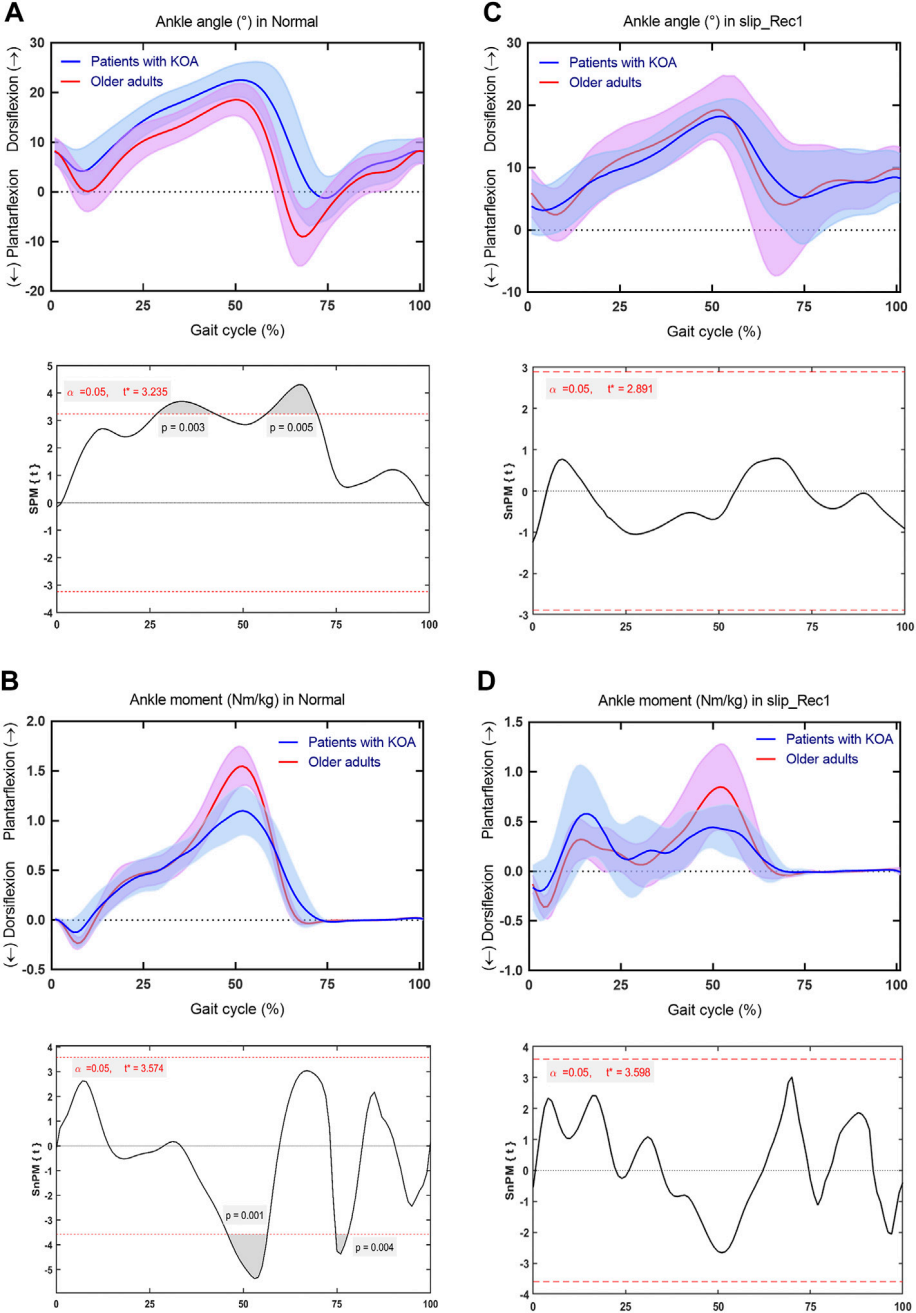
Comparison between patients with knee osteoarthritis (KOA) and older adults for the ankle angle and ankle moment in Normal and slip_Rec1 in the sagittal plane. Ankle angle for patients with KOA and older adults in Normal **(A)**, ankle moment for patients with KOA and older adults in Normal **(B)**, ankle angle for patients with KOA and older adults in slip_Rec1 **(C)**, and ankle moment for patients with KOA and older adults in slip_Rec1 **(D)**. The upper and lower panels present the average and standard deviation of patients with KOA and older adults and the corresponding outcomes of the one-dimensional statistical parametric mapping (SPM) scalar trajectory *t*-test, respectively. For each analysis, the significance level is set at 0.05, and the corresponding t* is presented as the horizontal red dashed line. The *p* values associated with the supra-threshold clusters, denoted as the grey shaded regions, are presented whenever the test statistic continuum SPM{t} or SnPM{t} exceeds the threshold.

At the ankle joint, for Normal, patients with KOA showed significantly larger dorsiflexion angles throughout 26.79%–42.6% of the gait cycle (*p* = 0.003) and smaller plantarflexion angles throughout 56.18%–69.91% of the gait cycle (*p* = 0.005) than older adults (Figure 5A); lower plantarflexion moments throughout 45.77%–56.41% of the gait cycle (*p* = 0.001) and higher plantarflexion moments throughout 74.67%–77.91% of the gait cycle (*p* = 0.004) (Figure 5B). Nevertheless, for slip_Rec1, there were no significant differences for ankle angle or moment between groups (Figures 5C,D). The ankle range of motion (ROM) is presented in Table 2, with no significant differences between the two groups for both Normal and slip_Rec1.

**TABLE 2:** Ankle, knee, and hip range of motion (ROM) in the sagittal plane.

Variable	Normal		*p-*value	Slip_Rec1		*p-*value
	Patients with KOA	Older adults		Patients with KOA	Older adults	
Ankle ROM (°)	25.96 ± 4.07 [22.84, 29.09]	28.69 ± 3.38 [26.82, 30.57]	0.0900	19.06 ± 4.66 [15.47, 22.64]	22.25 ± 6.99 [18.38, 26.12]	0.2380
Knee ROM (°)	51.34 ± 10.80 [43.04, 59.64]	61.86 ± 4.38 [59.43, 64.28]	0.0200*	42.01 ± 13.22 [31.85, 52.16]	55.45 ± 7.87 [51.09, 59.81]	0.0047**
Hip ROM (°)	37.61 ± 10.96 [29.19, 46.04]	43.64 ± 6.95 [39.80, 47.49]	0.1114	27.64 ± 10.17 [19.82, 35.46]	41.40 ± 10.32 [35.69, 47.12]	0.0043**

The data are presented as the mean ± standard deviation [95% Confidence Interval]. *p < 0.05, **p < 0.01. Abbreviations: KOA, knee osteoarthritis; Rec1, first recovery step.

### Knee Joint

**FIGURE 6:**
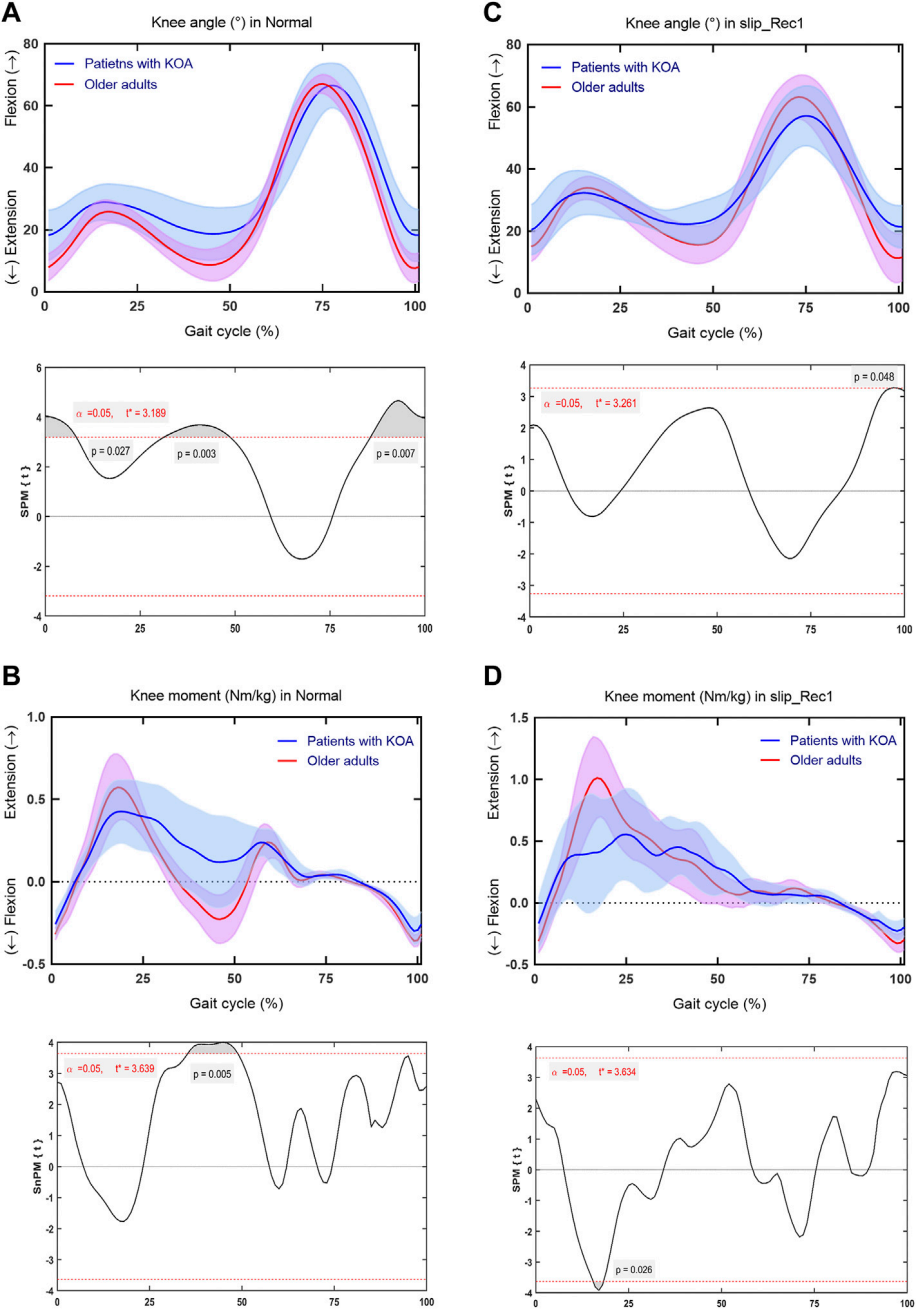
Comparison between patients with knee osteoarthritis (KOA) and older adults for the knee angle and knee moment in Normal and slip_Rec1 in the sagittal plane. Knee angle for patients with KOA and older adults in Normal **(A)**, knee moment for patients with KOA and older adults in Normal **(B)**, knee angle for patients with KOA and older adults in slip_Rec1 **(C)**, and knee moment for patients with KOA and older adults in slip_Rec1 **(D)**. The upper and lower panels present the average and standard deviation of patients with KOA and older adults and the corresponding outcomes of the one-dimensional statistical parametric mapping (SPM) scalar trajectory *t*-test, respectively. For each analysis, the significance level is set at 0.05, and the corresponding t* is presented as the horizontal red dashed line. The *p* values associated with the supra-threshold clusters, denoted as the grey shaded regions, are presented whenever the test statistic continuum SPM{t} or SnPM{t} exceeds the threshold.

Regarding the knee joint, for Normal, patients with KOA developed significantly smaller extension angles across 0%–8.188% (*p* = 0.027), 31.23%–48.69% (*p* = 0.003), and 85.63%–100% (*p* = 0.007) of the gait cycle (Figure 6A), smaller flexion moments across 35.30%–48.90% of the gait cycle (*p* = 0.005) (Figure 6B) than older adults. For slip_Rec1, patients with KOA only developed significantly smaller extension angles across 96.48%–98.18% of the gait cycle (*p* = 0.048) (Figure 6C) and smaller extension moments across 15.47%–18.23% of the gait cycle (*p* = 0.026) (Figure 6D). The knee ROM is presented in Table 2, with significant differences between the two groups for both Normal (*p* = 0.0200) and slip_Rec1 (*p* = 0.0047).

### Hip Joint

**FIGURE 7:**
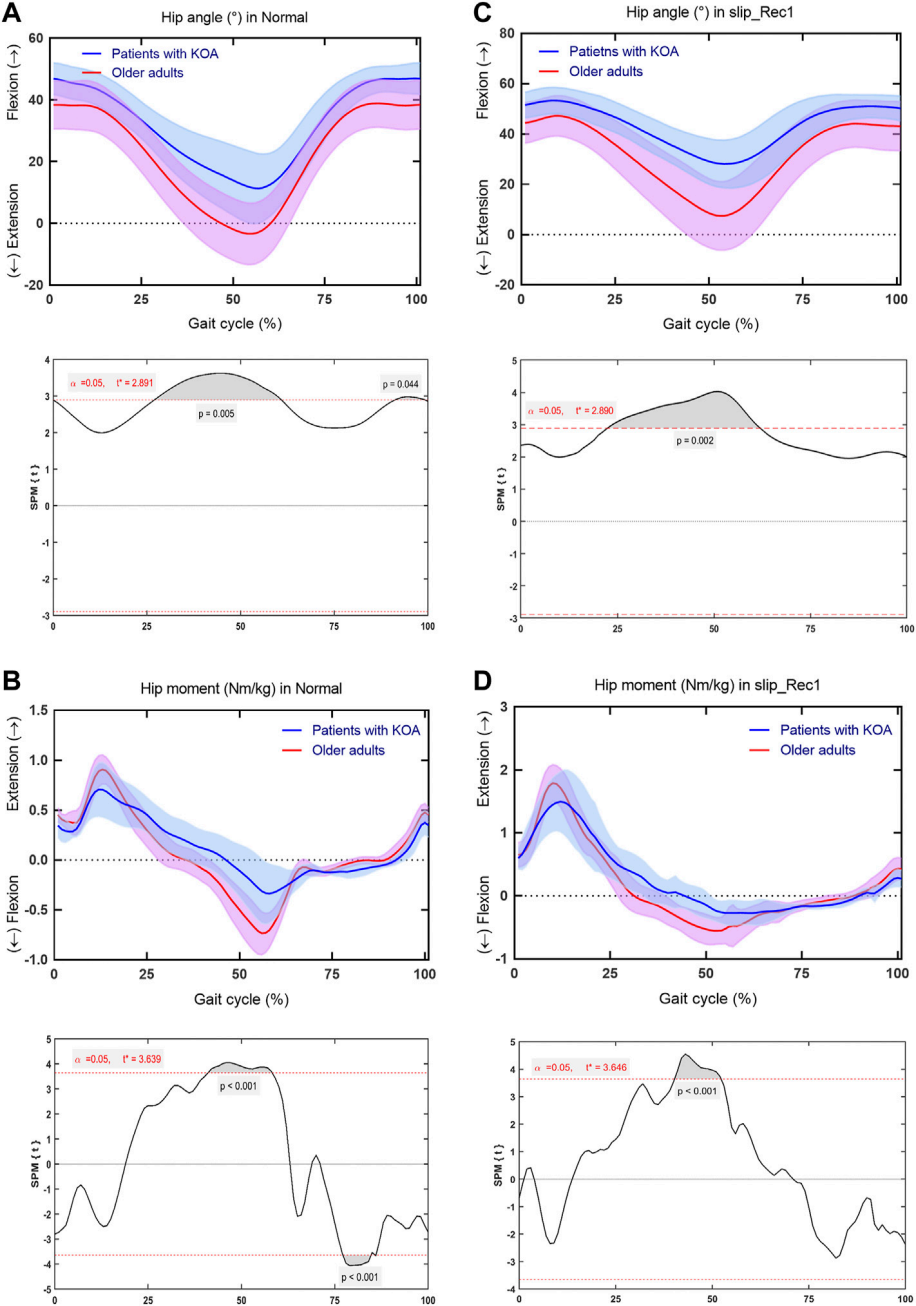
Comparison between patients with knee osteoarthritis (KOA) and older adults for the hip angle and hip moment in Normal and slip_Rec1 in the sagittal plane. Hip angle for patients with KOA and older adults in Normal **(A)**, hip moment for patients with KOA and older adults in Normal **(B)**, hip angle for patients with KOA and older adults in slip_Rec1 **(C)**, and hip moment for patients with KOA and older adults in slip_Rec1 **(D)**. The upper and lower panels present the average and standard deviation of patients with KOA and older adults and the corresponding outcomes of the one-dimensional statistical parametric mapping (SPM) scalar trajectory *t*-test, respectively. For each analysis, the significance level is set at 0.05, and the corresponding t* is presented as the horizontal red dashed line. The *p* values associated with the supra-threshold clusters, denoted as the grey shaded regions, are presented whenever the test statistics continuum SPM{t} or SnPM{t} exceeds the threshold.

At the hip joint, for Normal, patients with KOA showed significantly smaller extension throughout 26.98%–60.94% of the gait cycle (*p* = 0.005) and flexion angle throughout 91.18%–99.23% of the gait cycle (*p* = 0.044) (Figure 7A), smaller flexion moments throughout 41.04%–58.24% of the gait cycle (*p* < 0.001) and larger extension moments throughout 77.17%–84.87% of the gait cycle (*p* < 0.001) (Figure 7B). For slip_Rec1, patients with KOA also showed significantly smaller extension throughout 22.4%–62.01% (*p* = 0.002) (Figure 7C) and smaller flexion moment throughout 40.23%–52.26% of the gait cycle (*p* < 0.001) (Figure 7D). The hip ROM is presented in Table 2, with significant differences between the two groups for slip_Rec1 (*p* = 0.0043).

## Discussion

This study investigated the compensatory strategies between normal walking and the first recovery step following a backward slip perturbation in patients with KOA compared with age-matched older adults. The novelty of this study is that the adapted gait perturbation procedure induces the mechanism of human falls and quantifies the lower limb response to gait compensation after the onset of a slip perturbation. Our findings indicate that the pathological condition of patients with KOA limits the normal gait pattern compared with older adults. The spatiotemporal parameters, as well as joint kinematics and kinetics of the lower extremities, showed significant differences in response to the perturbed gait, confirming that patients with KOA have a higher risk of falling relative to healthy older adults. Based on the fact that no falls occurred, effective compensatory strategies in the first step following backward slip perturbations might act primarily through lower extremity joints to prevent falls.

In line with previous studies (Debi et al., 2012; Sun et al., 2017; Wiik et al., 2017), patients with KOA showed significantly shorter step lengths relative to older adults in Normal. In this regard, the step length of slip_Rec1 is considered to be a critical element for improving balance recovery after a perturbation, as sufficient step length may help restore balance and may be more effective in avoiding falls (Debelle et al., 2021). In the current study, the step length of slip_Rec1 decreased slightly compared with Normal, even though statistical significance was not reached between Normal and slip_Rec1 in either group. This indicates that the step length is controlled within the effective range after the occurrence of a slip perturbation. Milner et al. (2018) indicated that the purpose of shortening step length in patients with KOA is to reduce knee joint loading, thereby preventing falls (Milner et al., 2018). However, patients with KOA may be at greater risk of falling in response to perturbations relative to older adults, as there was also a significant difference in the step length of slip_Rec1 between the two groups.

Gait speed is a predictor of morbidity in patients with KOA (Alenazi et al., 2021); this factor is related to functional capacity in adults with mobility impairments (Peel et al., 2013). Gait speed reduction has been documented as a risk factor for falls in patients with KOA (Purser et al., 2012; Taş et al., 2014; Gill et al., 2017). In the current study, the gait speed of patients with KOA in Normal was significantly smaller than that of older adults, which is consistent with previous studies (Debi et al., 2012; Queen et al., 2016; Wiik et al., 2017; Vennu and Misra, 2018; Alenazi et al., 2021). The difference between the groups was still present in slip_Rec1, whereas the gait speed of slip_Rec1 did not differ from the Normal gait speed within the groups. This means that both patients with KOA and older adults are within their capabilities for speed control after a backward slip perturbation, and gait speed does not serve as an indicative indicator of their response to the perturbation but may be considered a predictor of increased fall risk in KOA patients compared to healthy older adults. Patients with KOA not only showed lower gait speed, but this also declined over time, even after controlling for BMI and other covariates (Vennu and Misra, 2018; Alenazi et al., 2021). However, in the present study, the difference in BMI between the two groups was significant (*p* = 0.001), which may have contributed to the decrease in gait speed, although no correlation analysis between the two was performed. A large population-based cohort study found that decreased gait speed was significantly and independently associated with BMI in patients with symptomatic KOA (King et al., 2018). On the other hand, knee ROM (Brinkmann and Perry, 1985), quadriceps muscle weakness (Spinoso et al., 2018), and quadriceps tendon stiffness (Ebihara et al., 2021) are factors that influence the gait speed of patients with KOA. Quadriceps tendon stretching (Ebihara et al., 2021) and resistance training (Li et al., 2021) may be effective in improving gait speed. As gait speed increases, step length also increases (Bovi et al., 2011; Fukuchi et al., 2019). Therefore, we strongly recommend that patients with KOA focus on strengthening the quadriceps muscles, regardless of the severity of KOA. This may also be an important part of improving gait stability.

GRFs are a screening tool for rapid detection of abnormal joint loading (Tang et al., 2004) and a key factor in predicting KOA severity (Kotti et al., 2014). Among patients with KOA, the significant reduction in push-off force and push-off impulse during the terminal stance phase makes GRFs abnormal, which leads to an overall decrease in gait speed (Wiik et al., 2017). In addition, it has been shown that vGRFs are lower in patients with severe KOA (Moustakidis et al., 2010). Our results showed that the normalized vGRFs of patients with KOA were significantly lower than that of older adults, regardless of whether it is in Normal or slip_Rec1. Kotti et al. (2014) found that vGRFs reached zero after approximately 73% of the gait cycle in healthy subjects, compared with approximately 71% in patients with KOA (Kotti et al., 2014). The end of the stance phase was the most distinctive difference between the healthy individuals and patients with KOA, with the stance phase being prolonged in healthy subjects compared with patients with KOA (Kotti et al., 2014). Our results appear to be contrary to the above-mentioned study, with the vGRFs reaching zero for older adults (66% in Normal and 65% in slip_Rec1) earlier relative to patients with KOA (71% in Normal and 67% in slip_Rec1). These findings corroborate a previous study (Spinoso et al., 2018). Patients with KOA remain in the stance phase for a longer time, suggesting that the prolonged effect of a minor overload could be comparable to the short effect of intense joint stress (Kirkwood et al., 2011). From a clinical perspective, the negative effects of the prolonged stance phase are joint overload and early fatigue, leading to a gradual increase in joint wear (Spinoso et al., 2018).

Concerning the joint ROM of the lower extremity, only the knee joint ROM was significantly different in both Normal and slip_Rec1. Restricted ROM of knee flexion appears to be an important determinant of locomotor disability in patients with KOA (Steultjens et al., 2000). Knee flexion ROM was lower in patients with KOA than in older adults throughout the stance phase in the present study, showing decreased flexion angles at the stance phases of loading response and terminal stance phase. This finding enriches the existing literature (Astephen et al., 2008; Zeni and Higginson, 2009; McCarthy et al., 2013). Likewise, the knee flexion moment was decreased and the knee extension moment was increased across the stance phase in patients with KOA, a finding that is comparable to the peak moment values in a previous study (Astephen et al., 2008). This behavior is considered to be a compensatory strategy used by patients with KOA in response to pain (McCarthy et al., 2013). A recent study reported that the activation effect of the quadriceps is improved with an increased knee joint extension moment (Hyodo et al., 2020), which plays a critical role in improving gait speed. This finding is supported by the significant difference between patients with KOA and older adults across the mid-stance phase for slip_Rec1. In our study, there were significant differences in the knee extension angle across the terminal swing phase for both Normal and slip_Rec1, but there were no significant differences of flexion moment. This is in line with a previous study in which the improvement in the knee was attributed to the overactivity of the rectus femoris muscle (McCarthy et al., 2013). From a biomechanical point of view, the strength of the quadriceps might be the main contributor to the difference between patients with KOA and older adults. In both Normal and slip_Rec1, the quadriceps muscle seems to be insufficient to fully extend the knee joint. Thus, it needs to be strengthened and activated effectively to increase the ROM of the knee extensor angles during walking. Therefore, effective training is needed to increase the ROM of the knee extensor angles. In this context, future studies using musculoskeletal multibody simulation are needed to examine the differences between patients with KOA and healthy older adults in quadriceps and other muscle forces (Tischer et al., 2017; Kebbach et al., 2020).

There were significant changes at the ankle angles, particularly across the mid and terminal stance phases and the pre- and initial swing phases in Normal, most notably as a marked lack of plantarflexion in patients with KOA. Correspondingly, for Normal, there were lower ankle plantarflexion moments across the terminal stance phase and the pre- and mid-swing phases in patients with KOA, findings that are consistent with a previous study on ankle plantarflexion moment extremes (Gonçalves et al., 2017). It has been shown that the use of ankle plantar flexors in the later stance phase of the gait cycle in patients with OA could lead to increase knee joint reaction forces (Fisher et al., 1997). Therefore, patients with KOA should try to avoid using ankle plantar flexors during gait to reduce the compressive force on the knee joint.

For Normal, there were significant differences in the hip joint involved in the mid/terminal stance phases and the pre- and terminal swing phases, whereas for slip_Rec1 the differences only involved the mid/terminal stance phases and the pre-swing phase. The main manifestation was an inadequate extension of the hip joint, leading to an insufficient hip flexion moment. Slip-induced falls could be exacerbated by an inability to generate sufficient joint moments (Chandler et al., 1990; Wojcik et al., 1999; Liu and Lockhart, 2009). Apparently, the muscles responsible for hip extension are primarily the gluteus maximus (Neumann, 2010; Alnahdi et al., 2012) and adductor magus (Neumann, 2010). A recent systematic review revealed that patients with KOA have significant hip strength deficits with hip abduction (Deasy et al., 2016); nevertheless, there is no clear result that indicates hip joint strength in the sagittal plane. It could be speculated from our results that adequate joint moments generated in the sagittal plane of the hip joint play a crucial role in both patients with KOA and older adults. This also confirms our hypothesis that relatively weaker hip muscle strength and inadequate activation account for the greater susceptibility to falls in patients with KOA compared with older adults.

Some limitations should be noted. First, the sample size of the KOA group in the current study is relatively small, with only nine patients including both males and females. Small samples may result in reduced statistical power, less reliability, and inflated effect sizes, which in turn may limit the general applicability of the findings (Mullineaux et al., 2001). However, a recent study suggests that a target power of 0.8 involving one-dimensional data effects could be achieved with small to moderate sample sizes (*n* = 5–40) in biomechanical experiments (Robinson et al., 2021). Therefore, we believe that our study should be feasible. For the follow-up study, we will recruit more patients with similar severity to further confirm our results. Second, we did not consider sex-induced differences in the investigated biomechanical parameters, since Phinyomark et al. (2016) found no significant differences in any discrete gait kinematic variables between KOA and healthy subjects by gender (Phinyomark et al., 2016). Furthermore, we did not directly analyze muscle forces acting in the lower extremity, which is an interesting aspect for future studies.

## Conclusion

Compared with healthy older individuals, patients with KOA have a higher risk of falling in response to a backward slip perturbation, which could be monitored effectively by key parameters such as hip angle, moment, knee ROM, and vGRFs. Patients with KOA should focus on the strength and activation of the muscles that play a major role in hip extension during gait from another perspective, i.e., the gluteus maximus and adductor magus, etc., and improve hip extension with specific exercises, such as targeted resistance training and perturbation-based stepping state training while focusing on knee-related quadriceps strength. This could be essential in addressing backward slip-induced gait perturbations.

## Data Availability

The raw data supporting the conclusion of this article will be made available by the authors, without undue reservation.
